# Recent Research Progress on Lignin-Derived Resins for Natural Fiber Composite Applications

**DOI:** 10.3390/polym13071162

**Published:** 2021-04-05

**Authors:** Bijender Kumar, Dickens O. Agumba, Duc H. Pham, Muhammad Latif, Dinesh, Hyun Chan Kim, Hussein Alrobei, Jaehwan Kim

**Affiliations:** 1 Creative Research Center for Nanocellulose Future Composites, Inha University, 100, Inha-ro, Michuhol-gu, Incheon 22212, Korea; bijenderkumarchem@gmail.com (B.K.); owinodickens@gmail.com (D.O.A.); phamduchoa.tdt@gmail.com (D.H.P.); mlatif8482@gmail.com (M.L.); dnshkmr477@gmail.com (D.); Kim_HyunChan@naver.com (H.C.K.); 2 Department of Mechanical Engineering, Prince Sattam Bin Abdul Aziz University, Al-Kharj 11942, Saudi Arabia; h.alrobei@psau.edu.sa

**Keywords:** lignin, epoxy resins, mechanical properties, flame retardancy, fiber composite

## Abstract

By increasing the environmental concerns and depletion of petroleum resources, bio-based resins have gained interest. Recently, lignin, vanillin (4-hydroxy-3-methoxybenzaldehyde), and divanillin (6,6′-dihydroxy-5,5′-dimethoxybiphenyl-3,3′-dicarbaldehyde)-based resins have attracted attention due to the low cost, environmental benefits, good thermal stability, excellent mechanical properties, and suitability for high-performance natural fiber composite applications. This review highlights the recent use of lignin, vanillin, and divanillin-based resins with natural fiber composites and their synthesized processes. Finally, discussions are made on the curing kinetics, mechanical properties, flame retardancy, and bio-based resins’ adhesion property.

## 1. Introduction

**Figure 1:**
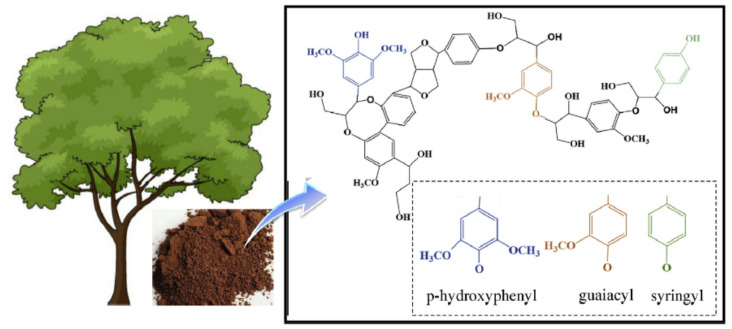
The chemical composition and structure of lignin. Adapted from Ref. [31], with permission from Elsevier.

Lignin is the second most abundant and high molecular weight natural phenolic polymer, which occurs from plant tissues. It contains a three-dimensional phenolic structure built up by *p-*coumaryl alcohol, coniferyl alcohol, and sinapyl alcohol [1], as shown in Figure 1. Although the industrial sector produces a large amount of lignin, around 95% every year, it is burned, resulting in severe resource waste [2]. Lignin has been used in limited industrial applications due to the complex structure with amphiphilic nature. Lignin is commonly soluble in dimethyl sulfoxide, but it also soluble in some organic solvents and aqueous organic solvent solution [3,4]. Despite the complex structure, lignin has recently attracted interest in many applications due to its inherent potential from its unique behaviors, such as antioxidants in plastics, scavengers, surfactants, and dispersants antibacterial, antitumoral, and adhesives [5,6,7]. Vanillin (VN) is the most well-known monoaromatic compound extracted from lignin at an industrial scale, although various compounds have been obtained from lignin [8,9,10]. At the industrial scale, the first time, the chemical depolymerization of lignin prepared VN in the presence of waste sulfite liquor. Most of the VN mainly extracted from wood, and it is commonly soluble in ethanol [11]. It is widely used in various applications such as cosmetics [12], pharmaceuticals [13], flavorings in foods [14,15], and as additive building blocks for polymer composites [16].

However, despite the broad use of lignin as a bio-based feedstock for materials, it shows incompatibility with other polymers and heterogeneity, leading to the deterioration of materials’ properties. Several studies have been reported to modify the lignin and enhance compatibility with polymers and mechanical properties for high-performance applications to overcome these drawbacks. Up to now, a large amount of research has been performed on bio-based thermoplastics such as bismaleimide/eugenol [17], poly(lactic acid) [18], bio-poly(ethylene terephthalate) [19], poly(butylene succinate) [20], polyhydroxyalkanoates [21,22], poly(ethylene 2,5-furandicarboxylate) [23], and also on thermosets such as itaconic acid [24], isosorbide [25], composite of cardanol novolac/bismaleimide [26], plant oil [27,28], eugenol [29], and polyesters [30]. On the other hand, average research progress has been published on preparing thermoplastic and thermosets resins with lignin. In contrast, much less progress has been made on the VN and divanillin (DVN)-based resin. The literature combining lignin-derived-based resins such as lignin, VN, and DVN is necessary to explore bio-based resin’s value in natural fiber composites application for years to come.

Bio-based thermoplastic and thermoset polymers are gaining attraction due to their biodegradability and environmental concern that can replace petroleum feedstock-based products [32,33]. Until now, several studies have described the use of lignin in biothermoplastics, including biocomposites due to its modifications possibility, availability, biodegradability, and good mechanical properties [6,34,35]. However, much less research progress has been made on the lignin, VN, and DVN-based thermoset resins compared to the thermoplastics. In engineering fields, thermoset resins are generally used in electronic components, adhesive, aircraft industry, and automotive because of their high strength, high modulus, good durability, good thermal stability, and chemical resistance [34,36]. Thermoset resins have a wide range of properties that depend on the curing cycle, hardener, and the proportions of hardener used during the preparation of thermoset resins [8].

Lignin, VN, and DVN-based resins, like thermoplastics and thermosets, are considered promising materials for natural fiber composites to increase the binding strength. Due to the low dimensional stability of lignin and natural fibers, it is necessary to study the development of lignin, VN, and DVN-based resins, which can be used for natural fiber composites. In this review article, research progress on thermoset resins preparation for natural fiber composites, especially lignin, VN, and DVN-based resins, was surveyed, and future research directions were suggested. Few review papers have indeed been published related to bio-based epoxy resins [37,38,39]. However, to our knowledge, no review paper of lignin, VN, and DVN-based resins has been published, which draws the research community’s attention to developing bio-based natural fiber composites. Finally, the objective of this review is to describe the critical futures of lignin-derived resins. Ultimately, this review’s literature combination can be utilized as a benchmark for future materials science communities, thereby encouraging them to develop fully bio-based natural composites materials for high-performance applications.

## 2. Lignin-Based Resins

**Figure 2:**
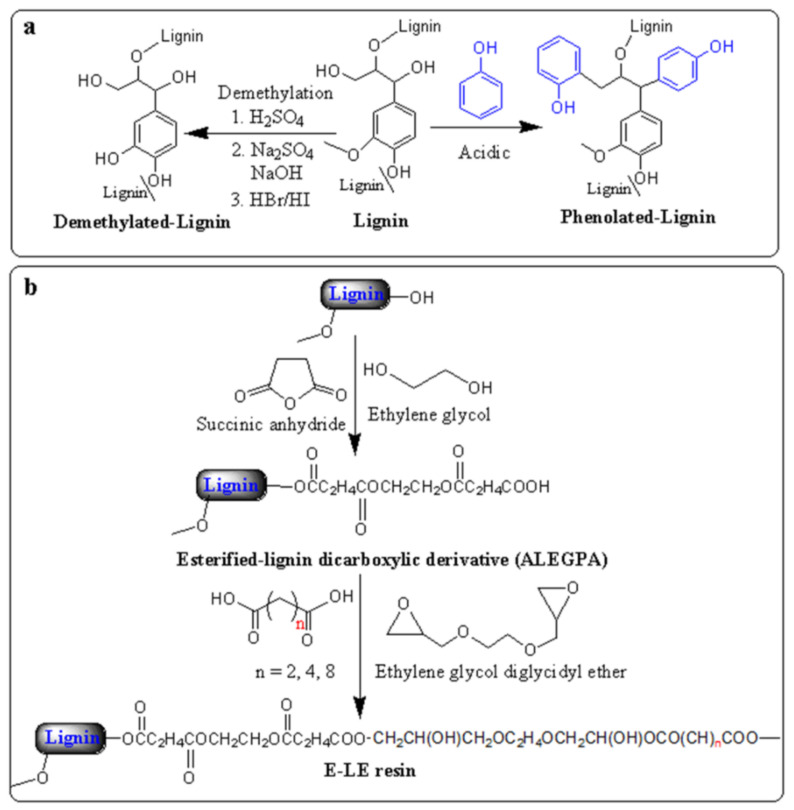
Synthetic diagram route of (**a**) demethylation and phenolization of the lignin and (**b**) ester-based lignin epoxy (E-LE) resin.

As a cross-linked natural polymer containing phenolic content and low-cost availability, Lignin has been studied for a broad range of applications [40,41]. It is produced from the pulp and paper industry around 70 million tons per year, but only 2% of it has been used to recover energy in the pulping industry, and the rest is uncommercialized waste materials [42]. As a bio-based material, it has been used in several applications. Nevertheless, owing to the decomposition of amorphous phenolic structure, higher char and tar formation during the conversion process, it is difficult to develop higher value materials [43]. Instead, the researcher’s attention has been increased due to lignin’s unique properties, such as low toxicity, biodegradability, ecofriendly, and sensitiveness to enzymatic degradation [44]. In recent years, lignin has been used with various polymers to prepare composites and epoxy resins for different applications, i.e., surfactants, coatings, lubricants, plasticizers, carbon fiber, wastewater treatment, stabilizing agents, and fire retardant [1,8,45,46,47]. Several studies have been described the phenolization/demethylation of lignin by using various types of catalysts such as H_2_SO_4_ [45], hydrogen bromide (HBr), hydrogen iodide (HI) [48], 1-dodecanethiol (DSH) [44], and Na_2_SO_3_ [49] to improve the reactivity and also to reduce the molecular weight of lignin for thermoset applications (Figure 2a) [45,50,51]. Since HI has strong nucleophile behavior, it is a more effective catalyst for lignin demethylation than H_2_SO_4_, DSH, HBr, and Na_2_SO_3_. They optimized the reaction parameters such as catalyst, temperature, and time to increase the phenolic hydroxyl group. Wang et al. also found that the HI catalyst depicts more reactivity than HBr, resulting in that HI has more capacity to demethylation of lignin [48]. They also stated that the adhesive properties of phenolated lignin were increased compared to lignin [52,53].

Hirose et al. [54,55] prepared the ester-based lignin epoxy (E-LE) resin by the alcoholysis reaction of lignin (AL) with succinic anhydride in the presence of ethylene glycol (EG), as shown in Figure 2b. The esterified-lignin dicarboxylic derivative, ALEGPA, was mixed with ethylene glycol diglycidyl ether and different dicarboxylic acid types. They investigated the effect of ALEGPA and dicarboxylic acid derivatives (succinic acid, adipic acid, and sebacic acid) content on the E-LE’s glass transition temperature (T_g_). T_g_ of E-LE resin increased with increasing of ALEGPA content due to the increasing cross-linking. In other words, lignin works as a hard segment in E-LE polymeric network. Succinic acid-based resin depicts higher T_g_ as compared to adipic acid and sebacic acid. T_g_ decreased with increasing the alkylene chain length attributed to the increasing molecular motion with alkylene chain length.

**Figure 3:**
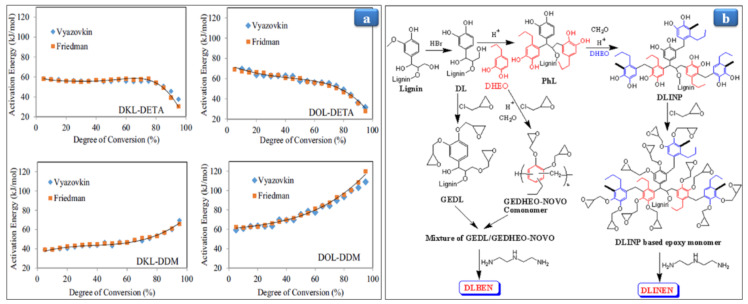
(**a**) The activation energy behaviors of the curing reaction versus conversion. Adapted from Ref. [56] with permission from Elsevier. (**b**) Schematic representation of demethylated lignin phenol-based epoxy monomer and resin.

Ferdosian and coworkers [56] have synthesized a depolymerized lignin epoxy resin by reacting the depolymerized Kraft lignin (DKL) and organosolv lignin (DOL) with epichlorohydrin in the presence of an alkaline medium. The curing agents prepared a cured epoxy resin, namely, aliphatic amine-like diethylenetriamine (DETA) and aromatic amine-like 4, 4′-diaminodiphenyl methane (DDM). The curing behavior of cross-linked lignin resin was investigated using differential scanning calorimetry (DSC) with various methods such as Vyazovkin [57], Friedman [58], and Kissinger [59], given the activation energy. The Kissinger method cannot clearly explain the curing mechanism and the activation energy of epoxy-amine due to the non-catalytic and autocatalytic reaction process, homopolymerization, and etherification [60,61,62]. In Vyazovkin and Friedman’s methods, two activation energy trends appeared for cured DKL-DDM, DOL-DDM, DKL-DETA, and DOL-DETA energy depend on the degree of curing conversion. In comparing cured DKL-DDM and DOL-DDM epoxy resins, the activation energy of cured resins DKL and DOL with DETA appeared approximately constant up to 0.7 degrees of conversion (α). It decreased gradually up to 30 kJ/mol due to the autocatalytic mechanism [60,61,62], as shown in Figure 3a. Several studies have reported the same trends for the activation energy [62,63,64]. On the other hand, for cured DKL and DOL with DDM, the activation energy in the initial stage (α value from 0 to 0.8) slightly climbed, and after that it quickly increased in the later stage due to the self-curing reaction pathway (Figure 3a). The same trend has been reported in several studies [60,61,62]. Moreover, cured DKL resin exhibited lower activation energy than those of the DOL resin due to more –OH groups in the DKL resin, resulting in a high curing process. In the case of DDM, the activation energy increases due to the no proper mixing between resin and hardener resulted in the self-curing process. In contrast, the activation energy trend for DETA occurred due to the autocatalytic mechanism. The DKL-DDM and DOL-DDM demonstrated relatively higher thermal stability than the DKL-DETA and DOL-DETA owing to the aromatic structure of DDM. Moreover, cured DKL with DDM resin depicted higher thermal stability than DOL resin (Table 1). Besides, all cured epoxy resins have the char residue in the range 23–38%, which is higher than conventional diglycidyl ether of bisphenol A (DGEBA). This result indicates that the DKL-DDM can compete with the petroleum-based epoxy resins.

Chen et al. [65] prepared the polyols-like depolymerized lignin (DL) with high hydroxyl content by depolymerizing alkali lignin at the optimized NaOH concentration (15 wt%) and temperature (250 °C). They reported that this method increases the –OH group, about 2.11% in DL, and reduces environmental pollution. NaOH’s effect, temperature, and time were investigated on the cured neat epoxy, demethylated lignin-based epoxy resin (DLEP), and original lignin-based epoxy resin (OLEP) as an epoxy value, thermal stability, and tensile strength. At NaOH (15 wt%), time (1.5 h), and temperature (250 °C), DLEP has a maximum epoxy value of 0.82% with higher tensile strength (2.66 MPa). Lignin plays a vital role in enhancing the thermal stability of the neat epoxy resin, and it increases the thermal stability of DLEP up to 258 °C. OLEP is 244 °C, while the cured epoxy resin shows thermal stability up to 240 °C, as shown in Table 1. Zhao et al. [66] have reported renewable epoxy networks; namely, deprotected lignin incorporated novolac epoxy networks (DLINEN), glycidyl ether of deprotected lignin (GEDL), glycidyl ether of dihydroeugenol (DHEO)-novolac oligomer (GEDHEO-NOVO), and deprotected lignin blended epoxy networks (DLBEN) as shown in Figure 3b. The effect of lignin content ratios was studied on the epoxy structure as cross-link density (ρ) and thermal stability. DLINEN-5 (5 wt% lignin content loading in DLINENs) exhibited the lowest ρ, around 9.45 × 10^−3^ mol/cm^3^, compared to the neat epoxy resin, namely, GEDHEO-NOVO, which attributed to the reactivity and steric hindrance of lignin. The DL-based epoxy network depicted higher ρ than the lignin-based epoxy network due to the reactivity and low molecular weight [67]. DLINEN-5 exhibited higher α-relaxation temperature (T_α_) around 141 °C, while the LINENs and LBENs depicted T_α_ in the range 130–137 °C, although T_α_ for that GEDHEO-NOVO is 139 °C. The higher T_α_ of DLINEN-5 is attributed to the increasing ρ, which restricts the polymeric chain mobility [68,69]. These results demonstrated that the lignin-based resins have T_α_ more than 130 °C near the T_α_ (137 °C) of DGEBA/DETA [70]. All modified lignin-based epoxy resins exhibited the first degradation in the range of 250–425 °C, corresponding to the aliphatic chain’s breaking. In contrast, the second degradation occurred at 425–650 °C due to breaking the aromatic moiety’s C–C bond [71].

**Figure 4:**
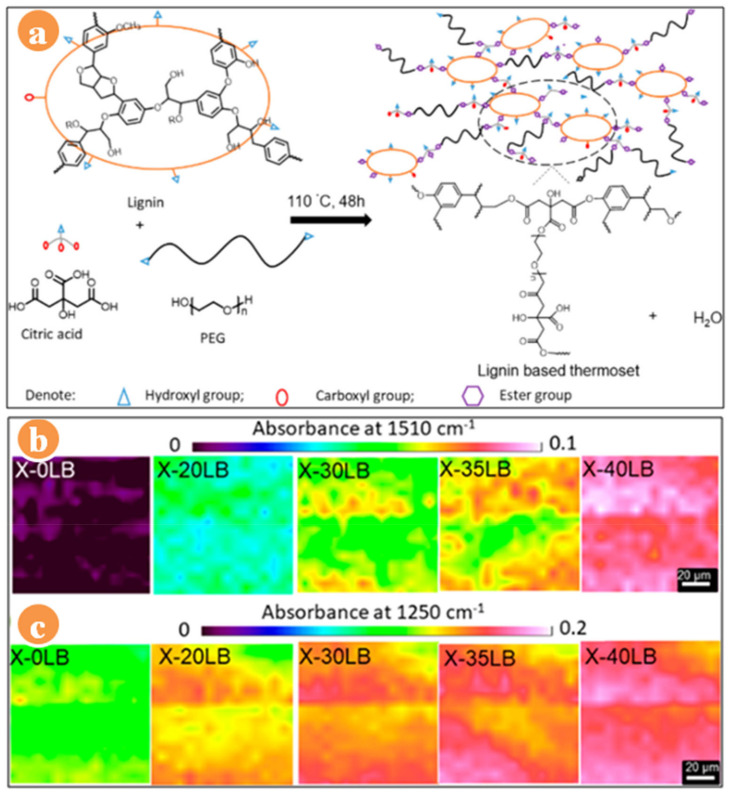
(**a**) Representative schematic crosslinking of lignin, (**b**) absorbance intensity patterns of aromatic content, and (**c**) ester bond [75].

Van de Pas et al. [72] have introduced the bio-based lignin epoxy resin monomer, prepared by reacting epichlorohydrin with wood, followed by wood’s hydrogenolysis in the presence of Pd/C [73,74]. The blend of hydrogenolysis epoxy prepolymer (LHEP) and oligomer lignin hydrogenolysis epoxy pre-polymer (LHOEP) with bisphenol A diglycidyl ether (BADGE) was prepared using curing agents, namely, isophorone diamine (IPDA) and DETA. The cured DETA-LHEP/BADGE blend at various compositions exhibited the T_g_ range from 68 to 80 °C. It decreased with the increasing content of LHEP, and the blend of LHOEP also depicted the same trend for T_g_, while the T_g_ of cured BADGE was 119 °C, which might be attributed to the rigid structure [70]. As increased the loading wt% of LHEP and LHOEP, static heat-resistant index temperature (T_s_) decreased due to the electron-donating –OCH_3_ groups of lignin (Table 1). In contrast, cured DETA-LHEP/BADGE and DETA-LHOEP/BADGE blends depicted better properties such as flexural modulus (3.7 GPa and 3.9 GPa), flexural strength (129 MPa and 163 MPa) than the BADGE resin. Based on the mechanical properties of the bio-based blends, these resins have comparable properties to the BADGE. Concerning toxicity and environmental issue, esterified lignin-based thermoset (PEG-CA-lignin) resin has been reported by the cross-linking of citric acid with lignin and poly(ethylene glycol) 400 (Figure 4a) [75]. The esterified resin’s degree of cross-linking increases with loading lignin content wt% ratios from 20 to 40. Figure 4b,c presents the distribution of lignin content and formation of the ester bond. At loading 40 wt% of lignin, the ester bond (around 1250 cm^−1^) distribution was higher than those of other feed ratios of lignin. It means the degree of cross-linking density and content of rigid aromatic are increased in thermosets-based resin. Besides, tensile strength was enhanced from 1.2 to 34.3 MPa with loading wt% of lignin; however, elongation at break decreased to 6% ± 2%. It might be occurred due to the increasing content of rigid aromatic resin. At loading wt% of lignin from 20 to 40, the thermosets resin demonstrated the higher T_s_ around 151 °C compared to the without loading lignin content. It occurred due to the cross-linking density of lignin with PEG and CA [75].

**Figure 5:**
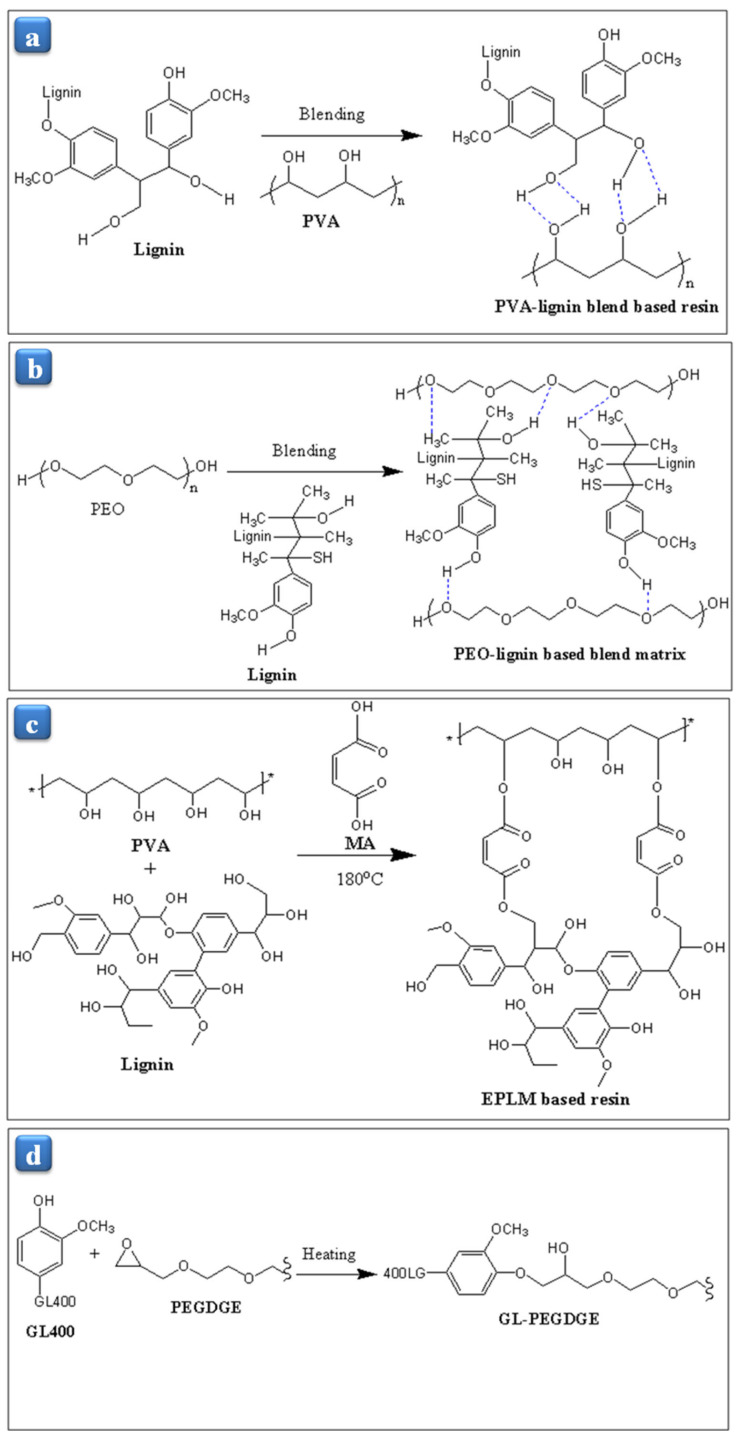
Schematic reaction diagram for the development of (**a**) PVA-lignin resin, (**b**) PEO-lignin blend matrix, (**c**) esterified PVA-lignin-maleic acid (EPLM) resin, and (**d**) elastomeric GL-PEGDGE resin.

Ko and coworkers [35] reported the ecofriendly polyvinyl alcohol (PVA)-lignin resin, and it was prepared by blending lignin with PVA’s various weight ratios from 10 to 50 wt% in deionized (DI) water (Figure 5a). It was observed that above 30 wt% of PVA content with lignin was able to form the film. PVA-lignin resin demonstrated thermal stability up to 230 °C. The 1st weight loss occurred around 70 °C due to the water dehydration and elimination. The 2nd weight loss happened in the range of 230–500 °C. Young’s modulus of the PVA-lignin resin decreased from 3.44 to 2.48 GPa with the increasing PVA content from 20 to 50 wt% because PVA has a low Young’s modulus. However, PVA-lignin resin’s toughness nearly ten times increased from 60.7 to 600.1 kPa, associated with increasing the tensile strength and elongation at break (Table 1). In this regard, Jayaramudu et al. [31] have developed the poly(ethylene oxide) (PEO)-lignin blend resin by blending lignin with PEO (Figure 5b) and investigated the effect of the lignin content on the thermal stability and mechanical properties. They found that the PEO-lignin blend resin’s thermal stability increased with increasing the lignin content from 10 to 30%. Young’s modulus of the 30% PEO-lignin was appeared 2.5 times higher than the neat PEO while the yield strength was 1.4 times. On the other hand, the elongation at break increased with increasing the lignin content by up to 20%. It decreased after that due to the decreasing pore size of the PEO-lignin blend resin. Recently, Ko et al. [34] have prepared the esterified PVA-lignin-maleic acid (EPLM) resin by the mixing of maleic acid (MA) from 0 to 50 wt% with PVA and lignin (1:1 ratios) as shown in Figure 5c. They optimized the esterified temperature of reaction 180 °C to better mechanical properties and found that the –OH peak at 3490 cm^−1^ was merged with the –OH peak of PVA-lignin resin. Moreover, the thermal stability of EPLM resin was slightly higher, around 240 °C at 20 wt% of MA, than the PVA-lignin resin (230 °C) [35], but when the weight ratios of MA increased from 20 to 50 wt%, the thermal stability decreased due to the non-reacting content of MA. As increasing the MA ratio from 10 to 40 wt%, the tensile strength, Young’s modulus, and toughness rose from 14.7 to 48.5 MPa, 1.7 to 2.4 GPa, and 78.2 to 787.7 kJ/m^3^, respectively. Although PVA-lignin resin had a tensile strength, Young’s modulus, and toughness of 41.1 MPa, 2.5 GPa, and 600.1 kJ/m^3^, respectively (Table 1) [35]. Moreover, at 50 wt% of MA, the mechanical properties decreased due to the MA molecule’s unreacted content. The observation concluded that the EPLM resin could compete with the commercial epoxy resin. Recently, Ono and coworkers [76] have reported the elastomeric epoxy resin, and it was prepared by mixing poly(ethylene glycol) diglycidyl ether (PEGDGE) and poly(ethylene glycol)-modified lignin (GL400) as a curing agent as shown in Figure 5d. The resin curing was performed at 100 °C that was below the curing temperature of 130 °C. GL400 was isolated from the Japanese cedar softwood by using acid-catalyzed solvolysis [77,78]. The resin’s GL content examined with the help of NMR, and the results demonstrated that if the GL content was more than 71 wt%, the resin could not be cured due to higher viscosity, and it was not uniformly mixed. Note that the elastomeric resin occurred when the GL content was over 50 wt%, and below 50 wt%, GL-epoxy resin did not successfully cure. The effect of GL content investigated on the mechanical and physical properties of GL-PEGDGE resin, and it observed that the hardness of the resin achieved like rubber hardness at 71–55 wt% of GL400 contents, and also the tensile modulus and tensile strength increased with increasing the GL content. On the other hand, the elongation at break increased similarly to the hardness, and it reached its maximum at 58.8 wt% of GL content. The results illustrated that the GL400 epoxy resin was expected to be used for structural matrix material and sealing materials.

## 3. VN-Based Resins

**Figure 6:**
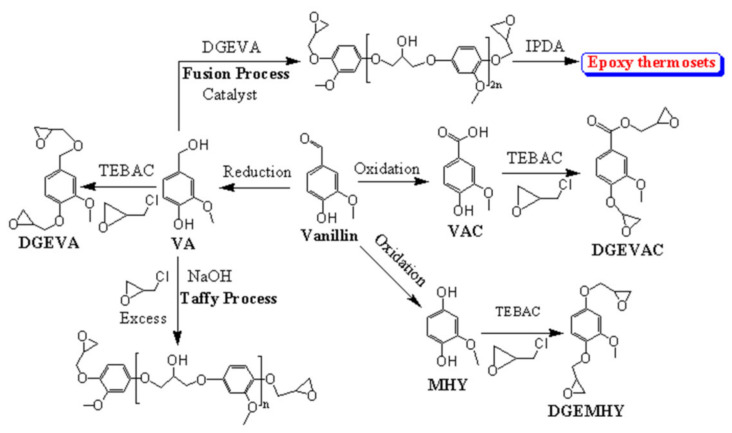
An illustration schematic reaction of epoxy monomers and epoxy thermosets polymer.

**Figure 7:**
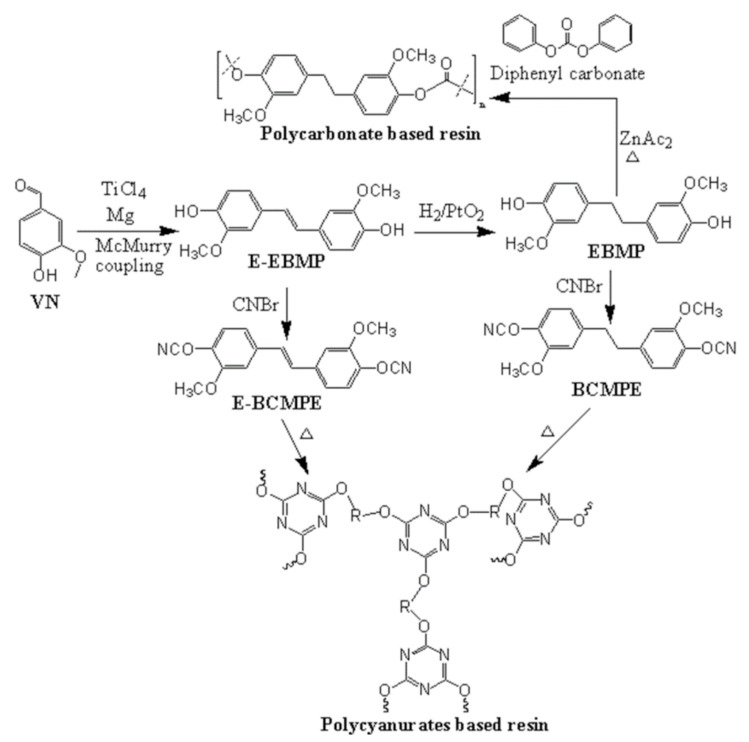
An illustration schematic reaction of cyanate-based monomers, polycyanurates, and polycarbonate-based resin.

VN (aromatic compound) is a suitable bio-based resin that can compete with petroleum-based resins. It is also using for pharmaceutics, fragrance, and flavor [79,80,81]. Around 85% of VN is prepared by synthetica methods from petroleum-based materials, like phenol, and the rest 15% occurs from lignin. VN is prepared through the alkaline and oxidative process of lignosulfonates followed by the sulfite pulping of wood, in which 55% of VN was prepared by hydrolysis, and the rest produced by oxidation [16,82]. For VN-based resins’ market size, synthetically derived VN demand was around 16,000 tons per year in 2012 [83], which increases day by day. It will reach 18,000 tons in 2016 [84] to about 50,000 tons in 2024 [85]. As an attractive bio-based epoxy monomer, VN has been incorporated into various polymers to enhance their properties for use in broad commercial applications [86,87,88]. Fache and coworkers reported multiple types of bio-based VN epoxy monomers, as shown in Figure 6 [11,89,90]. They synthesized the monomer by oxidation or reduction of VN, followed by the epichlorohydrin, carbonation, allylation, alkylation, and amination of VN derivatives such as methoxy hydroquinone (MHQ), vanillic acid (VAC), and vanillyl alcohol (VA). To oligomerization of diepoxy monomers, they also studied the taffy and fusion processes for industrial scale. It found that the fusion process is better than the oligomers’ taffy process at the industrial scale due to low cost, solvent-free, and simple purification methods. So, cured epoxy thermosets were synthesized by the fusion method using the equal ratios of VA and diglycidyl ether of vanillyl alcohol (DGEVA), followed by the curing in the presence of IPDA [89]. On the other hand, the cured materials of epoxy monomers such as diglycidyl ether of methoxy hydroquinone (DGEMHQ), diglycidyl ether of vanillic acid (DGEVAC), DGEVA, and vanillin-based oligomers [89] were prepared by the curing of IPDA, as an amine-based hardener. They investigated the effect of structure monomer and ρ on the properties of bio-based and cross-linked polymers. It was found that the DGEMHQ showed the highest T_g_ (132 °C) at a 2:1 epoxy/amine ratio [90]. As increased the amine ratio from 1.0 to 2.0, the T_g_ of DGEMHQ decreased to 78 °C, which might be due to the trapped excess amine or epoxy inside the polymeric matrix. DGEVAC exhibited higher T_g_ (152 °C) and T_α_ (166 °C) than the DGEMHQ (T_g_ = 132 °C, T_α_ = 154 °C) and DGEVA (T_g_ = 97 °C, T_α_ = 106 °C) due to the conjugation of the ester group with the aromatic ring, leading to more rigidity and higher ρ of DGEVAC. DGEVA has methylene carbon, which provides flexibility and rotation of the molecules, result in low T_g_. Moreover, DGEMHQ has fewer carbon atoms than DGEVA, resulting in less rotation, which makes it rigid. Harvey et al. [91] have reported the cyanate-based monomers namely, (E)-1,2-bis(4-cyanato-3-methoxyphenyl)ethene(E-BCMPE) and 1,2-bis(4-cyanato-3-methoxyphenyl)ethane (BCMPE). E-BCMPE was synthesized by the McMurry coupling reaction of VN, followed by the reaction of (E)-4,4-(Ethene-1,2-diyl)bis(2-methoxyphenol) (E-EBMP) and cyanogen bromide (CNBr). On the other hand, BCMPE was prepared by the hydrogenolysis step of E-EBMP, including the reaction of CNBr and 4,4-(Ethane-1,2-diyl)bis(2-methoxyphenol) (EBMP) (Figure 7). The polycyanurates thermosets were prepared from the cyanate-based monomers under thermal curing. Meanwhile, the polycarbonates-based thermoplastic resin was prepared by the transesterification of diphenyl carbonate and BCMPE, as shown in Figure 7. The thermally cured BCMPE resin showed T_g_ to be 202 °C, comparable to commercial petroleum-based resins, while the polycarbonate-based resin exhibited the T_g_ around 86 °C.

**Figure 8:**
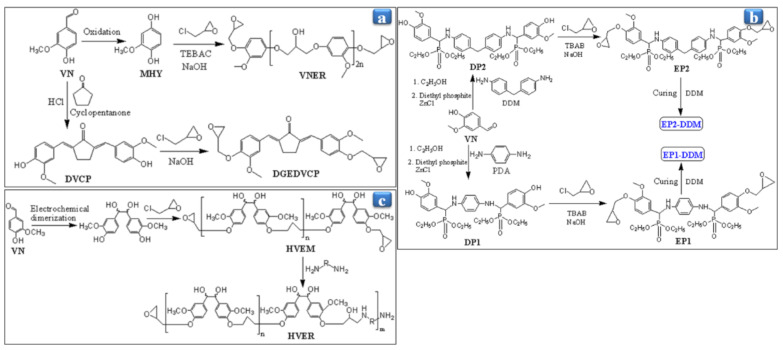
An illustration schematic reaction of (**a**) VNER and DGEDVCP, (**b**) EP1-DDM and EP2-DDM, and (**c**) HVEM and HVER.

Recently, Nikafshar et al. [33] have synthesized the bio-based VN epoxy resin (VNER) through VN’s oxidation with sodium percarbonate, followed by the epichlorohydrin of MHY (Figure 8a). A cured VNER was prepared by curing of inorganic accelerator (IA) as a curing agent with epoxy monomer and Epikure F205. They have also reported in their previous work that the inorganic accelerator (calcium nitrate) is suitable to reduce the curing temperature and time of the epoxy resins [92]. They studied the curing behavior of DGEBA/Epikure F205 (EF205), VNER/EF205, and VNER/EF205/2 wt% IA resins with the help of DSC analysis. It found that the curing process temperature of VNER/EF205/2 wt% IA was divided into two steps: for the first step, the initial onset temperature (T_1_) and the first peak temperature (T_p1_) were 8.5 °C and 26.65 °C, respectively, while for the second step 50.1 °C and 71.2 °C, respectively. For DGEBA/EF205, T_1_ and T_p1_ were 55.8 °C and 93.1 °C, while for VNER/EF205 60.1 °C and 97.0 °C, respectively. DSC analysis demonstrated that VNER/EF205/2 wt% IA’s curing process started earlier than the without inorganic accelerator and uncured epoxy resin. This curing behavior of resins has been reported [93,94]. DGEBA/EF205 depicted a higher T_g_ (52.1 °C) than the VNER/EF205. Meanwhile, the 2 wt% IA increased the T_g_ of VNER/EF205 from 38.3 to 47.9 °C due to the polymeric chain’s mobility restriction [95,96,97]. Based on mechanical properties, the cured VNER/EF205 with 2 wt% IA can compete with the DGEBA resin because it showed higher tensile strength than the VNER/EF205 and DGEBA resin (Table 2). Owing to IA’s presence, the polymeric chain of VNER/EF205/2 wt% IA is changed from an unspecified configuration to a parallel configuration. It means the polymeric chain becomes linear in IA’s presence, resulting in higher tensile strength than the DGEBA resin.

Further, Shibata et al. [98] have synthesized the VNER, as shown in Figure 8a, by the crossed-aldol condensation of cyclopentanone and VN reaction, followed by the transformation of the 2,5-bis(4-hydroxy-3-methoxybenzylidene)cyclopentanone (DVCP) into the required epichlorohydrin. The diglycidyl ether of DVCP (DGEDVCP) was cured with various hardeners, such as quercetin (QC), phenol novolac (PN), and guaiacolnovolac (GCN). They optimized the hardeners’ effect on the properties of DGEDVCP, and it found that the DGEDVCP-QC exhibited the loss modulus (E″) peak temperature to be 130 °C, which was near to the DGEDVCP-PN (128 °C), but it was around 82 °C for DGEDVCP-GCN. The higher E″ of DGEDVCP-QC was due to QC’s rigid structure and five –OH groups than PN, although GCN has lower –OH functionality than PN. Additionally, QC hardener produced more cross-linked resin with high tan δ around 141 °C than those of PN (131 °C) and GCN (100 °C). Besides, DGEDVCP-PN showed the storage modulus (E′) to be 1.64 GPa compared to DGEDVCP-GCN (1.29 GPa), E′ of DGEDVCP-QC (1.06 GPa) was near to the cured DGEBA resin due to the steric hindrance of epoxy and hydroxyl groups of QC. Moreover, DGEDVCP-PN demonstrated a higher thermal degradation temperature at 5 wt% than DGEDVCP-QC and DGEDVCP-GCN (Table 2). The flexural strength and moduli of all synthesized VNER were comparable with the cured DGEBA resin. Based on mechanical properties, DGEDVCP-PN resin is promising material and can be suitable for replacing petroleum-based resin. Wang and coworkers attached the phosphorus-based moiety to the environment-friendly VN-based resin thermosets to enhance the flame retardancy (FR) of epoxy thermosets [8]. The FR-based thermosets were synthesized by coupling reaction of diamines, such as 4, 4-diaminodiphenylmethane (DDM) and p-phenylenediamine (PDA) with VN followed by transformation resulting in the DP1 and DP2 into the required epichlorohydrin, afterward curing EP1 and EP2 monomers in the presence of a hardener (Figure 8b). Cured EP1-DDM and EP2-DDM depicted the higher flame retardancy (FR) than that of the cured DGEBA because EP1-DDM and EP2-DDM depict the higher limit oxygen index (LOI) to be 31.4% and 32.8%, while for that to DGEBA-DDM was 24.6% (Table 2). VN-based resins showed the higher char residues to be 35.8% and 41.2% than the DGEBA-DDM due to the remaining portion of phosphorus in the char yield, which prevents the heat transfer [99,100]. The higher T_g_ of cured EP1 (183 °C) and EP2 (214 °C) occurred due to the aromatic rigidity and intramolecular hydrogen bonding [101,102,103], although flexible phosphate groups are present in the polymeric chain. At the same time, the T_g_ for DGEBA-DDM was 166 °C. Relative to the DGEBA-DDM, the EP1 and EP2 resins showed low thermal stability at T_d5%_, which might be attributed to the O=P-O bond’s degradation [104]. Although at T_d30%_, these resins showed higher thermal stability due to the phosphorus-containing moiety that works as a thermal insulating in the epoxy matrix [100,105]. Meanwhile, EP2-DDM has higher phosphorus content than the EP1-DDM, resulting in higher thermal stability at T_d30%_ than EP1-DDM. EP2-DDM exhibited a higher modulus compared to EP1-DDM due to the rigid aromatic ring. On the other hand, the elongations at break of EP2-DDM (2.6%) and EP1-DDM (5.2%) were lower than those of DGEBA-DDM (7.4%), which might be due to the higher cross-link density and rigidity, leading to the lower mobility of the polymeric chain segment. EP resin showed outstanding mechanical properties like tensile strength (80.3 MPa) and tensile modulus (2.7 GPa) than the cured DGEBA. Amarasekara and coworkers synthesized the hydrovanilloin epoxy monomer (HVEM) using the electrochemical dimerization of VN, followed by the transformation of epichlorohydrin with NaOH (Figure 8c) [106]. Moreover, the hydrovanilloin epoxy resin (HVER) was prepared by the curing of various aliphatic amines such as 1,2-diaminoethane (1,2-DAE), 1,4-diaminobutane (1,4-DAB), 1,6-diamino hexane (1,6-DAH), and IPDA. Cured HVER-1,6-DAH showed the higher T_g_ to be 149 °C than those of other resins, namely, HVER-1,2-DAE (116 °C), HVER-1,4-DAB (118 °C), and HVER-IPDA (146 °C). The higher T_g_ of HVER-1,6-DAH might be attributed to the better packing of hardener in the polymeric chain [107]. Moreover, the hardness of HVER increased with increasing the number of the carbon atom of curing agents.

**Figure 9:**
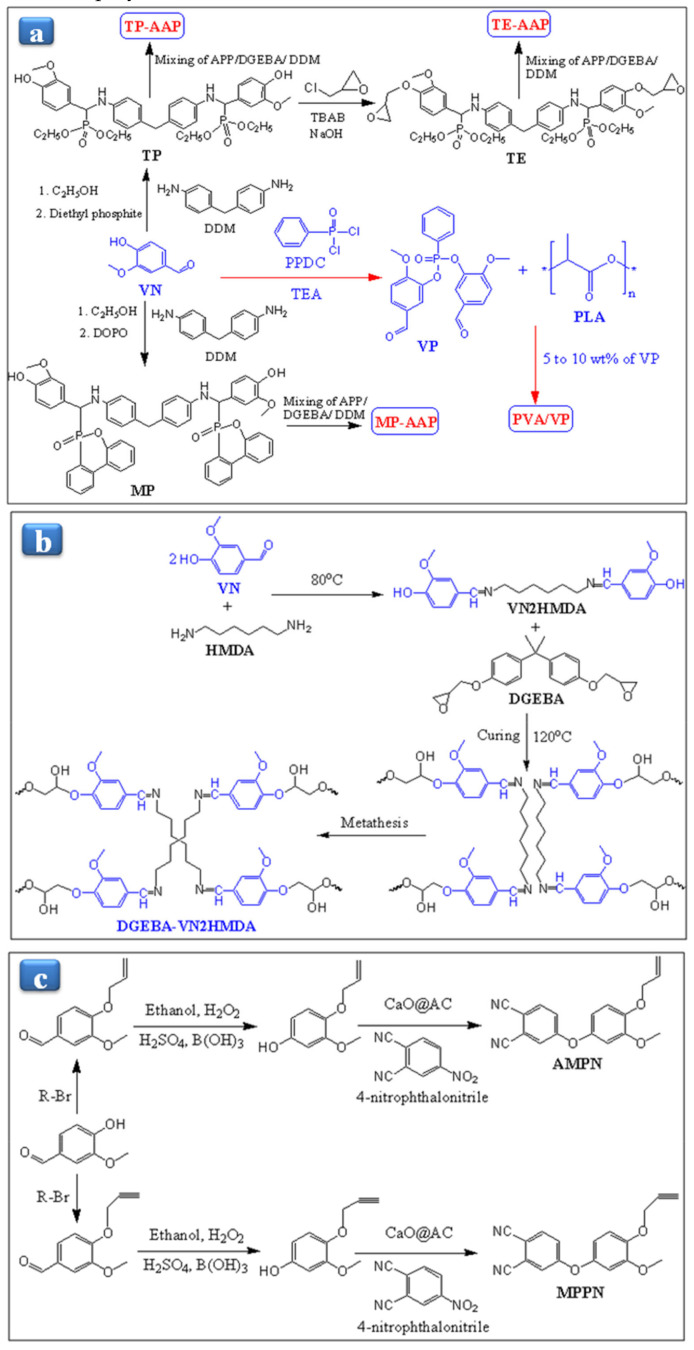
An illustration reaction scheme for the synthesis of (**a**) TP-AAP, MP-APP, TE-APP-based materials, and PVA/VP composite, (**b**) VN2HMDA, and curing reaction mechanism of DGEBA and VN2HMDA, and (**c**) AMPN and MPPN.

Xu et al. [108] have synthesized the TP, MP, and TE resins using ammonium polyphosphate (APP) as an FR enhancer to increase the FR efficiency of VN-based resins (Figure 9a). They studied APP content’s effect on the Tg, FR, and thermal stability of VN-based materials. They found that the T_g_ of the cured TE-APP decreased from 176 to 153 °C with increasing the APP concentration, and it also decreased with increasing the TE content. Moreover, TE0-APP15 resin showed a better FR with LOI value (35.4%) and the UL-940 test. At the total ratios of 10 phr of TE and APP, the LOI values of the TE-APP resin reached up to 29%. On the other hand, the T_g_ of MP-APP increased from 164 to 176 °C, decreasing MP and APP’s total ratios from 10 to 5 phr. The increased T_g_ of MP-APP might be due to the cross-link density of polymers [109]. Meanwhile, TP-APP resin depicted the lowest LOI value due to the lower cross-links density because TP resin does not have an epoxy group in the polymeric chain. Moreover, the MP3-APP7 described the better LOI value of 29.4% with excellent FR, which can be attributed to the polymeric chain’s phosphorus group [110,111]. The thermal stability of VN-based resins was calculated using the equation [112] with TGA results.

(1) $$T_{s}=0.49\left[T_{d5\%}+0.6\left(T_{d30\%}-T_{d5\%}\right)\right]$$

Here, T_s_ denotes the statistic heat resistant index and T_d5%_ and T_d30%_ are at 5 wt% and 30 wt% loss in TGA. The TGA results demonstrated that the T_s_ of the TE-APP resin was near the MP-APP resin, while the T_s_ for TP-APP resin was the lowest. Therefore, TE-APP resin depicted the highest thermal stability among the resins. On the other hand, MP-APP resins showed good thermal stability due to the rich benzene ring. The thermal and FR properties of TP, MP, and TE-based resins are summarized in Table 2.

To increase the FR and toughness, Cheng et al. [88] have prepared the bio-based VN/polylactic acid (PLA) composite by incorporating different ratios of bis(5-formyl-2-methoxyphenyl)phenylphosphonate (VP) into the PLA matrix. The VP was synthesized by the reaction of phenylphosphonic chloride (PPDC) and VN in the presence of TEA (Figure 9a). They investigated the FR and mechanical properties of VP content on the PLA/VP composite. PLA depicted the LOI value around 21.4%, which means PLA has flammable behavior, while the LOI values of VN/PLA composite reached from 21.4% to 26.3%, with the increasing weight ratio of VP from 5 to 10%. This result indicated that the VP is a potential FR enhancer material. They also performed the cone calorimeter test (CCT) of the composite to evaluate the FR. It was observed that the time to ignition (TTI) values of PLA and PLA10VN were around 68 s and 64 s, respectively. PLA5VP and PLA10VP were around 76 s and 79 s, which means that they have more FR behavior than the PLA and PLA10VN. PLA/5VP composite depicted the higher elongation at break and impact strength than those of pure PLA, which might be due to VP’s lubricant behavior, leading to an increase in composite chain mobility. On the other hand, Young’s modulus (3.5 GPa) and tensile strength (54 MPa) of PLA/5VP were similar to Young’s modulus (3.7 GPa) and tensile strength (57 MPa) of PLA. Relative to the VN, VP showed the T_d5%_ and T_max_ at 286 °C and 343 °C, respectively. PLA exhibited T_d5%_ and T_max_ at 326 °C and 363 °C, respectively. As increasing the wt% of VP, the composite’s thermal stability was found in 324–333 °C. The T_max_ increased from 366 to 380 °C. These results suggest that the VP is a considerable additive for plastics. The thermal and FR properties of PLA/VP-based composites are presented in Table 2. Mai and coworkers [80] prepared the ecofriendly and cross-linked DGEBA-VN2HMDA resin from the DGEBA resin by curing Schiff base dihydroxylimine hardener like VN2HMDA in the presence of 2-ethyl-4-methylimidazole (EMI) as a catalyst. Schiff base hardener was synthesized by the reaction of VN and hexamethylene-1,6-diamine (HMDA), as shown in Figure 9b. In the first heating, cured DGEBA-VN2HMDA resin exhibited one exothermic peak. However, no additional exothermic peak appeared in the second heating, indicating the complete curing between DGEBA and the hardener. The cross-linked DGEBA-VN2HMDA depicted the T_g_ as 78 °C, while the tan delta exhibited the T_g_ around 87 °C. The lower T_g_ of DGEBA-VN2HMDA resin may be due to the flexible segments of the hardener. On the other hand, the resin’s thermal stability also appeared lower (302.9 °C) than the conventional epoxy polymer due to the amine group’s low binding energy. DGEBA-VN2HMDA resin did not dissolve in common solvents in comparing uncured resin, but it swelled and afterward partially dissolved some content in a polar solvent. DGEBA-VN2HMDA has hydrolysis properties due to the imine bond, although it did not change the weight and shape with water immersion.

Recently, Han et al. [113] have synthesized the VN-based phthalonitrile monomers, namely 4-(4-(Allyloxy)-3-methoxyphenoxy)phthalonitrile (AMPN) and 4-(3-Methoxy-4-(prop-2-yn-1-yloxy)phenoxy)phthalonitrile (MPPN), by reacting VN and allyl/propargyl bromide, followed by the oxidation and phthalonitrile (Figure 9c). They studied the curing parameters and processability of phthalonitrile monomers with the help of DSC. The higher melting temperature (T_m_) of MPPN (130.4 °C) may be attributed due to the rigidity of propargyl content and higher viscosity (0.45 Pa.s) as compared to AMPN, which have T_m_ = 94.6 °C and viscosity = 0.08 Pa.s. Besides, the gelation temperature of MPPN was 286.2 °C, while that of AMPN was 294.6 °C. DSC revealed the three exothermic peaks after the T_m_ for both monomers. According to both monomers’ chemical structures, the first exothermic peak told in DSC might be due to the possibility of Claisen rearrangement [114]. The second peak was appeared due to self catalyzation reaction between the hydroxyl group and phthalonitrile moiety. However, the peak temperature and enthalpy were different for both monomers. The third exothermic peak in DSC was attributed to the self-polymerization. In the case of MPPN, the self-polymerization was not completed until 400 °C, while for AMPN, it was completed up to 380 °C. In the case of AMPN, the FTIR peak of alkenes (1639 cm^−1^) disappeared. The band intensities of –CN (at 2228 cm^−1^) and phthalocyanine (1014 cm^−1^) were reduced after curing at 375 °C, which means that the curing of AMPN ended the phthalocyanine stage [115,116]. For cured MPPN, peaks at 3270 cm^−1^ and 2131 cm^−1^ related to alkynes stretching were disappeared entirely. The hydroxyl group band did not appear, while the bands of –CN, phthalocyanine, isoindoline (1701 cm^−1^), and triazine (1350 cm^−1^) are remarkably reduced. This change indicates that the alkynes were completely rearranged but partially cross-linked. The calculated processing window temperatures for AMPN and MPPN were 185 °C and 148 °C, which is the difference in the gelation temperature and T_m_ temperature. Moreover, for the VN-based phthalonitrile monomer, this temperature was different from the petroleum-based monomers [117]. Regarding the processing window temperature for the cured AMPN, the first stage occurred from 200 to 280 °C due to the [3, 3] sigma rearrangement, and the second stage was above 280 °C, which was attributed to the polymerization of phthalonitrile. The third stage occurred at 330 °C, attributed to the self-polymerization. Regarding the curing process for MPPN, the first stage appeared due to the sigma rearrangement of propargyl aryl ether followed by the [1,5] hydrogen shift and electrocyclic ring-closing. On the other hand, the second stage process (280–330 °C) for curing was different from the AMPN, while the third stage started above 350 °C due to self-polymerization not completed up to 375 °C. In summary, based on FTIR and DSC analysis, they designed the processing window temperature program for both monomers from 170 to 375 °C. DMA measurement demonstrated that the cured MPPN depicted the lowest heat resistance and lower storage modulus (*E*′). However, T_g_ of the cured phthalonitrile resins was higher than 500 °C, comparable to the petroleum-based polymers. Navarukiene and coworkers [86] have synthesized the VN acrylate-based resin by the photo-initiated polymerization of vanillin diacrylate (VD) and vanillin dimethacrylate (VDM) in the presence of ethyl(2,4,6-trimethylbenzoyl)phenylphosphinate (TPOL) as an initiator. They studied the initiator (1, 3, and 5 mol% of TPOL) on the cured polymers as rigidity and polymers’ rigidity related to the storage modulus (Gʹ). In the series without solvent, VD-TPOL (3 mol%) showed higher Gʹ around 18.1 MPa in within 6s than VD-TPOL (1 mol%) and VD-TPOL(5 mol%), while Gʹ of VD-TPOL (3 mol%) reached up to 11.3 MPa within 12 s in the presence of a solvent, which means the solvent decreased the cross-linking rate due to the chain transfer agent [118]. In the case of VDM resin, the Gʹ value for VDM-TPOL (5 mol%) was higher in the presence of solvent than VDM-TPOL (1 mol%) and VDM-TPOL (3 mol%), which was due to more cross-linking on the surface layer and the slow polymerization [119,120].

## 4. Divanillin-Based Resins

**Figure 10:**
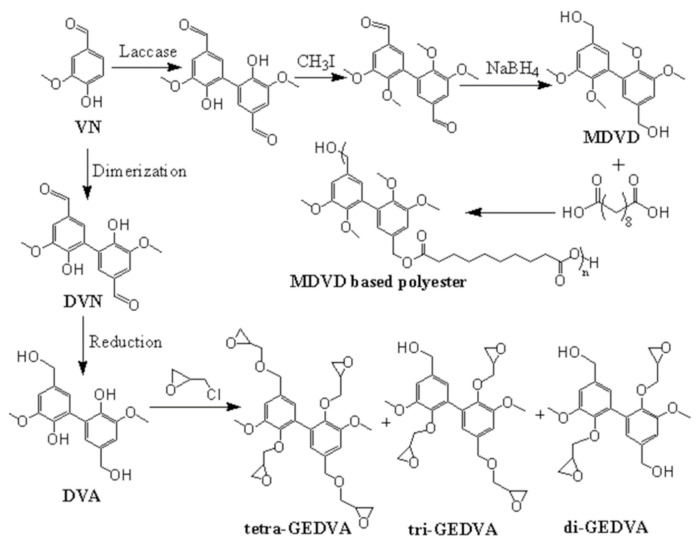
Representative reaction scheme for the development of MDVD-based polyester and GEDVA resin.

DVN is attractive as a sustainable and bio-based polymer derived from renewable resources like lignin. It is also considered a building block for polymer synthesis [121,122]. Several studies have reported the synthesized route of DVN by the oxidative phenol-coupling [123,124,125], enzymatic pathway [126,127], and dimerization of VN [128,129,130]. DVN is commonly soluble in ethanol. First times, DVN was synthesized by the oxidation of VN in the presence of hydrogen peroxide. Llevot et al. [126,127] have synthesized the methylated divanillyl diol (MDVD) monomer by the dimerization of VN in the presence of a laccase catalyst followed by the methylation and reduction. MDVD-based polyester was produced by the esterification of diols and diacids, as shown in Figure 10. Savonnet et al. [131] have developed the DVN epoxy resin, namely, tetra-GRDVA-IPDA, tri-GEDVA-IPDA, and di-GEDVA-IPDA using the dimerization of VN, followed by the reduction of aldehyde compound and epoxidation of divanillyl alcohol (DVA). The curing of bio-based glycidyl ether of divanillyl alcohol (GEDVA) monomer was performed with IPDA, as shown in Figure 10. They investigated NaOH/OH ratios’ effect on the yield%, T_g,_ and T_α_ of DVN epoxy resin. It found that the yield percentage of tetra-GEDVA to be 90% at 10 eq. NaOH/OH ratios. The thermoset behavior of the cured DVN epoxy resin monitored by DSC was based on the stoichiometric ratio (r), calculated by the equation [132].

(2) $$r=\;\frac{f_{\mathrm{epoxy}}\times n_{\mathrm{epoxy}}}{f_{\mathrm{hardener}}\times n_{\mathrm{hardener}}}$$

where f denotes the functionality and n denotes the molar quantity. At the ratio of r = 1, GEDVA-IPDA resin leads toward a thermoset resin because the r parameter plays the most important role between –NH moiety and epoxy [132]. DSC thermograms, when r = 1, demonstrated two exothermic peaks in which the first one at 100 °C and the second one at 140 °C for GEDVA-IPDA. Moreover, the second peak was not showed when r = 0.5. This change appeared due to the reactivity difference between the primary amine of IPDA and the secondary amino moiety formed during the curing [133,134]. Cured tetra-GEDVA-IPDA depicted the high T_g_ (198 °C) and T_α_ (200 °C) when r = 1; for di-GEDVA-IPDA, T_g_ = 138 °C, T_α_ = 140 °C; for tri-GEDVA-IPDA, T_g_ = 163 °C, T_α_ = 177 °C; and for DGEBA-IPDA, T_g_ = 152 °C, T_α_ = 155 °C. T_α_ increased from 140 to 200 °C due to the increased cross-linking with the epoxy content increase. Thus, epoxy networks led to reduced mobility. Nevertheless, the onset temperature of cured GEDVA-IPDA resin was lowest than that of DGEBA-IPDA, which means that GEDVA resin has a high reactivity tendency towards the N-H group. In comparing DGEBA-IPDA, GEDVA-IPDA resin exhibited the higher Young modulus, which increased from 1.45 to 1.9 GPa with the epoxy content increase. On the other hand, elongation at the break of DGEBA-IPDA was higher than those of GEDVA-IPDA resin due to the isopropyl network, which provides more flexibility to the DGEBA segment resin. The thermal degradation behavior of GEDVA-IPDA and DGEBA-IPDA was investigated in the view of T_s_ by Equation (1). GEDVA-IPDA resin exhibited two types of degradation; the first degradation occurred at 275 °C with 40% weight loss and the second near 500 °C with 60% weight loss, while for DGEBA-IPDA, the first degradation started near 330 °C with 70% weight loss. On the other hand, in N_2_, GEDVA-IPDA resin revealed the first degradation near 275 °C, while for DGEBA-IPDA near 350 °C. GEDVA-IPDA resin showed a lower T_s_ value around 20 °C than DGEBA-IPDA, attributed to the –OCH_3_ group’s degradation in an aromatic ring [135,136]. DVA epoxy resin has been known to be valuable alternatives to the DGEBA resin.

**Figure 11:**
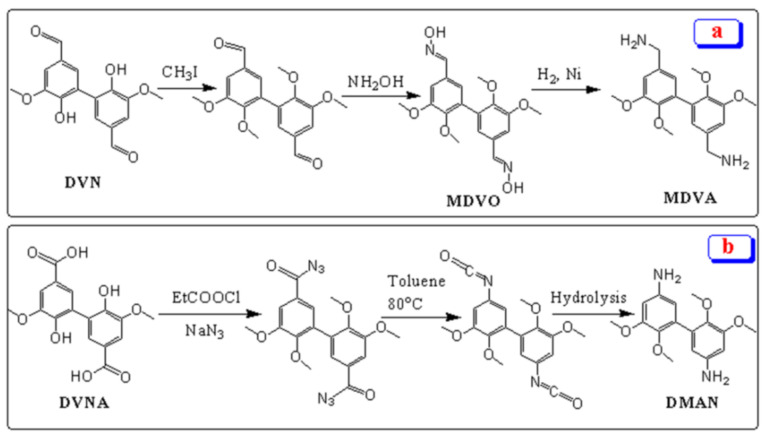
An illustration reaction scheme of (**a**) MDVA, and (**b**) DMAN.

Recently, in this way, Savonnet and coworkers [137] have reported the synthetic route for the development of DVN-based curing agents, namely methylated divanillylamine (MDVA) and 3,4-dimethoxydianiline (DMAN). MDVA was synthesized from the alkylation of DVN, followed by the oxidation of the aldehyde group, as shown in Figure 11a. DMAN was synthesized by the Curtius rearrangement, followed by hydrolysis of di-isocyanate (Figure 11b). These diamines, such as MDVA and DMAN, were used as curing agents at a stoichiometric ratio r = 1 to develop epoxy thermosets such as tetra-GEDVA-DMAN. Compared with DGEBA-4,4′-diaminodiphenyl sulfone (DDS) resin (T_g_ = 204 °C, char residue = 16%), tetra-GEDVA-DMAN exhibited the higher T_g_ around 212 °C with 48% char yield, which might be attributed to the C-C bonding between two aromatic rings in DMAN.

## 5. Applications of Lignin-Based Resins

**Figure 12:**
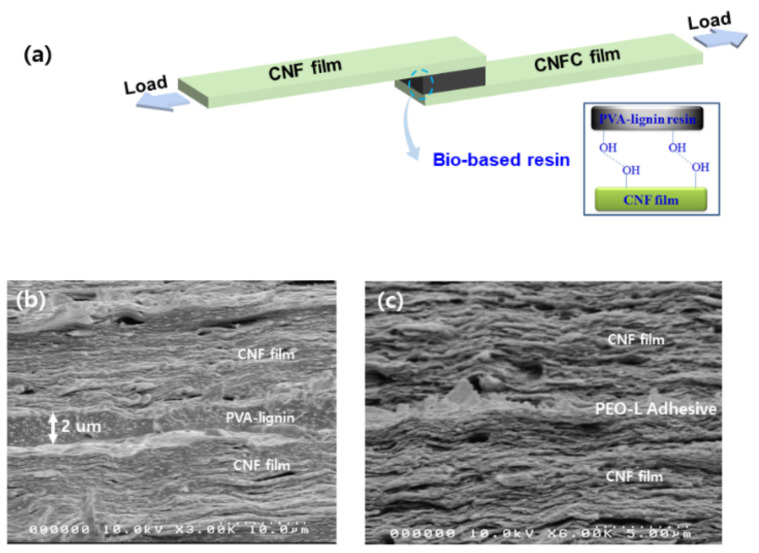
An illustration of the shear lap joint test of bio-based resins with CNF films (**a**) and cross-sectional SEM images of the specimens for PVA-lignin (**b**) [35] and PEO-lignin resins (**c**) [15].

Commonly, polymeric resins are categorized into epoxy and ester ones, in which ester resins are utilized for low-performance applications while epoxy resins for high performance [30,36,138,139]. Epoxy resins have more adhesion tendency to the surface of glass and carbon fibers than ester resins. Therefore, they are used for high-performance applications [140,141]. Compared to synthetic resins, recently, bio-based resins aim to develop thermosets, thermoplastic, and lightweight materials with better mechanical properties and replace commercial petroleum-based resins safe for environmental and healthcare concerns. Due to higher properties, bio-based resins’ demand is more increasing day by day than before [33,37,142,143]. Wood, a naturally higher strength fiber-reinforced composite, consists of lignin, cellulose, and hemicelluloses. Lignin plays the resin role as a waterproof and thermostability, while cellulose contributes as reinforcement [34]. Due to the thermostability and adhesive properties, the lignin-based resin has been suggested for fiber composite applications [31,34,35]. Several researchers have reported the lignin-based resin namely, hemp-epoxy [144], lignin/poly(ethylene oxide) [31,145], lignin/PVA [34,35], DL/epoxy [146], lignin/phenol formaldehyde [40,147], and fiber-reinforced composites [148]. Lignin-based resins have been used in broad applications such as sports equipment, tennis racquets, airplane, boats, and construction components for buildings due to their lightweight, high specific modulus and strength. Benjamin and coworkers [144] investigated the lignin weight percent effect into the hemp-epoxy composite to improve the fiber-to-matrix adhesion’s structural properties. The hemp-epoxy composite was developed by a vacuum-assisted resin transfer molding, in which epoxy was used as resin while hemp was used as reinforcement fiber. They found that 2.5 wt% of lignin is the optimized condition to improve Young’s modulus and ultimate tensile strength. Still, more than 2.5 wt% of lignin, these properties decreased due to the excessive portion of lignin particle that prevents the complete wetting from the fiber reinforcement. It was observed that the interfacial adhesion of 2.5 wt% lignin-based composite was better than those of 1.0 wt% lignin-based composite due to increasing the viscosity during the infusion. On the other hand, the pull out of the composite increased at 5.0 wt% lignin due to some porosity of composite [149]. Based on structural properties, the lignin hemp-epoxy composite is applicable for commercial products. Liu et al. [150] developed the lignin-based toughen epoxy resin and investigated alkali lignin carboxylic acid (AL–COOH) content effect on toughness reinforcement of the resin. At 1.0 wt% of AL–COOH, the resin’s fracture toughness is better than the neat epoxy resin. The lignin epoxy resin exhibited the roughen fracture surface, but the neat epoxy had a smooth surface with some river-like lines. It concluded that the AL–COOH was incorporated into the epoxy resin at the molecular level. The toughened lignin epoxy resin is a biorenewable candidate of coating material for high-performance composites. Lignin-based epoxy composite is developed through the loading of DL-epoxy into the cured epoxy networks [146]. It was observed that DL-epoxy was successfully incorporated into the epoxy network in the form of dark dots. These dots occurred due to DL-epoxy’s aggregation, caused by strong π–π stacking and hydrogen bond interaction. The fracture toughness of DL-epoxy/cured epoxy composite was evaluated using a critical stress intensity factor (K_1c_). K_1c_ increased with increasing the fraction of DL-epoxy in the composite. At 1.0 wt% of DL-epoxy fraction, the composite showed the highest K_1c_ near to 1.78 MPa.m^1/2^. Conversely to neat epoxy, DL-epoxy/cured epoxy exhibited low T_g_ due to the low cross-linking density at 1.0 wt% of DL-epoxy fraction [151]. The toughness depends on the feed ratios, cross-linking density, and T_g_ [152,153,154]. As compared to the straight fracture lines of neat epoxy, DL-epoxy/cured epoxy composite showed an irregular fracture surface with a large surface area. It indicates that the higher energy consumption for crack shows a more ductile nature than neat epoxy. Overall, after the incorporation of DL-epoxy, the fracture toughness, tensile strength, and ductility improved. Besides, its approaches are useable for composites application as additives. Ferdosian et al. studied lignin-based epoxy’s composites to investigate the flexural and adhesion strength properties. As compared to petroleum-based epoxy’s composites, lignin-based epoxy’s composites showed the lower flexural strength. In contrast, as increased the loading wt% of lignin-based epoxy matrix into the fiber-reinforced plastics, the composites demonstrated the nearest flexural modulus with petroleum-based matrix. Additionally, the adhesion strength of composites improved as the loading wt% of DOL-epoxy in the epoxy resin containing DGEBA increased. The loading 50 wt% of DOL-epoxy’ composites showed higher adhesion strength around 7.7 MPa (Table 3). The results of lignin-based epoxy resin indicated that it is a bio-based candidate for the high-performance application as a coating materials [155]. To this regard, Kraft lignin (KL) based resin was prepared by the cross-linking of glycerol diglycidyl ether (GDE) to reduce the utilization of formaldehyde-based resin. It can play a significant role as an adhesive at the industrial scale because it can be easily produced from various sources. The lignin-based adhesive was applied to the plywood to fabricate the composites and optimized the resin’s adhesion strength between plywood layers. The adhesion strength is increased with the increasing cross-linker ratios. At loading smaller particle size (>37 μm) of lignin in the resin, the resin showed better adhesion strength than higher particle size (>250–500 μm) of lignin. Note that the higher adhesion strength owing to the more cross-linking because smaller particles expose higher –OH groups for cross-linking. As compared to commercially based plywood samples that made by formaldehyde based resin, KL-GDE based resin depicted the better adhesion strength around 2.0–2.5 MPa (Table 3). The results indicates that KL-GDE resin’s performance is comparable to the formaldehyde-based resins [156]. In this regard, Ko and coworkers [35] have reported the PVA-lignin resin, and its adhesive property and toughness to cellulose nanofiber (CNF) film was investigated by the lap-shear joint (LSJ) test (Figure 12a). PVA-lignin resins of 50 wt% showed comparable properties with epoxy resin. After the LSJ test, PVA-lignin resin remained on both CNF films, which appeared due to the enhanced interaction between CNFs and PVA-lignin resin. It was observed that the shear strength increased with the increasing wt% of PVA fraction. At 50 wt% PVA, PVA-lignin resin exhibited the higher toughness (0.6 MPa) with high shear strength (4.4 MPa) (Table 3). Figure 12b,c showed the cross-sectional SEM images of PVA-lignin and PEO-lignin SLJ test specimens. Jayaramudu and coworkers [31] have prepared the PEO-lignin blend resin for the CNFs film. They evaluated the effect of lignin fraction on the blend’s adhesive property by using the SLJ test. Relative to the shear strength of pure PEO (0.44 MPa), PEO-lignin resin exhibited shear strength of 0.84 MPa at 30 wt% lignin content (Table 3). The SEM result demonstrated that the CNF films were jointed with PEO-lignin resin through hydrogen bonding (Figure 12c), and it could be used as an adhesive for fiber application.

Recently, Ko et al. [34] have reported the esterified PVA-lignin-maleic acid (EPLM) resin. The waterproof, toughness, and adhesive behavior of EPLM resin were investigated at various wt% of MA fraction. The water contact angle increased with the increasing wt% of the MA fraction, which indicates the hydrophobicity of resin. Note that the hydrophilicity nature of materials affects the adhesion properties because if resins have hydrophilic nature, which means they can absorb moisture, and resulting in the adhesion may deteriorate between the substrate surface and resins [157,158,159]. The failure strength of EPLM resin increased with the rising wt% of MA fraction, and it was found that the EPLM resin showed the highest failure strength (6.8 MPa) at 40 wt% of MA, whereas the failure strength for PVA-lignin resin was 4.4 MPa (Table 3). The higher toughness, hydrophobicity, and adhesive behaviors can be applied for natural fiber reinforcement applications.

## 6. Applications of Vanillin-Based Resins

**Figure 13:**
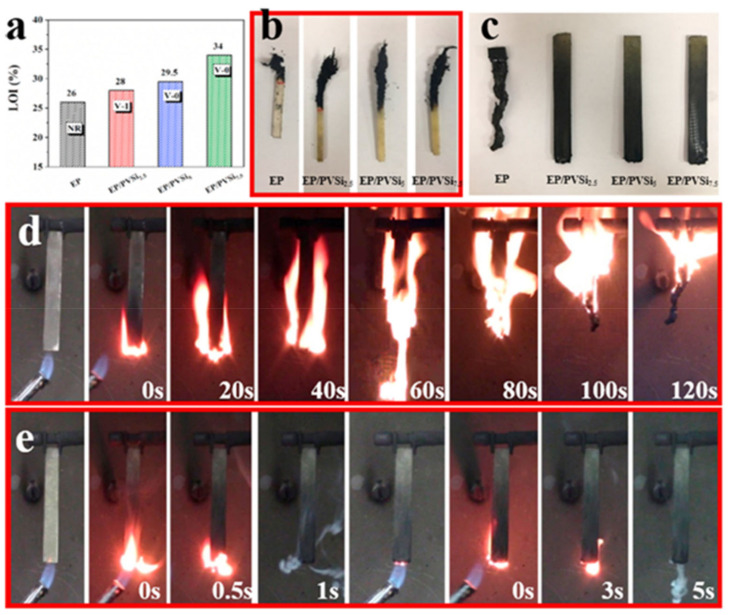
Limit oxygen index (LOI) and UL-94 test (**a**) epoxy matrix (EP) and EP-based composites, (**b**) the residue image of EP and EP-based composites, (**c**) UL-94 vertical burning test, digital photos of (**d**) neat EP, and (**e**) EP/PVSi-5.0. Reprinted with permission from F. Chu et al. [165] Copyright 2020 Elsevier.

Vanillin is an aromatic bio-based compound, and due to its aromatic character, its epoxy resins have been identified to replace vinyl ester-based resins [83]. Incorporating the aromatic moiety in the cured resins provides high thermal stability, resistance, and high strength because the aromaticity inhibits the polymeric matrix’s rotational freedom [11,33]. Many researchers have reported VN loading into various polymers for broad applications such as commodity plastic, adhesive, and flame retardants [8,88,160]. Epoxy resins are brittle and poor resistance matrix due to more cross-linking. Thus, epoxy resin research’s main challenge has been to increase the toughness of the epoxy resins. Several researchers have incorporated vanillin-based agents into the epoxy matrix to control the cross-link density to increase the toughness [80,88,161]. This attempt has been used to act as a toughening material for thermosetting polymers [88,150]. Nikafshar et al. [33] synthesized a tougher material such as VNER/EF205/2 wt%-IA. Relative to DGEBA (pull-off strength = 4.4 MPa), VNER/EF205/2 wt%-IA showed the pull-off strength of 3.7 MPa that indicates the adhesive property near to the DGEBA. Although, VNER/EF205/2 wt%-IA demonstrated higher pull-off strength than VNER/EF205 (1.37 MPa). It means the use of IA in the resins depicts positively impact pull-off strength in the materials. At loading 2 wt% of IA in epoxy resin, the Izod impact strength significantly increased up to 29.6 kJ m^−2^ compared to DGEBA (27.3 kJ m^−2^) because IA fills the gap of the epoxy matrix. Additionally, VNER/EF205/2 wt%-IA demonstrated the jagged and rough surface than those of DGEBA and VNER/EF205. It means VNER/EF205/2 wt%-IA can absorb higher energy, resulting in higher energy to break the materials, which indicates its higher toughness than the DGEBA [162]. This result suggested that it could be applied as a coating material for the high-performance application. Wang and coworkers [8] synthesized the flame retardant of VNER, namely, cured EP1-DDM and EP2-DDM that have similar reactivity to DGEBA. Moreover, these have higher FR ability with higher char yield, while DGEBA is a completely flammable material. The higher tensile strength, T_g_, and char yield of VNER indicated that it could be used in high-performance applications. Xu and coworkers [108] have reported the flame retardant environment-friendly VNER-based, such as TP-AAP, TE-AAP, and MP-AAP. Relative to DGEBA, VNER showed the higher FR with UL-49V0 and LOI (35%) at the Phr 10 of epoxy monomer and AAP. The T_g_ of TP-AAP, TE-AAP, and MP-AAP are comparable with DGEBA. Due to the higher char yield, these showed the potential use in high-performance materials. Wang and coworkers [163] reported the high-performance VN Schiff-based epoxy composite, i.e., CF/MB-PACM, which was prepared by the mixing of 3-methoxy-4-(oxiran-2-ylmethoxy) benzaldehyde(MB) and 4,4′-Methylenebiscyclohexanamine (PACM), followed by coating it on the carbon fiber (CF). CF/MB-PACM composite exhibited tensile strength (81 MPa) and T_g_ (172 °C) near the commercial CF/DER331-PACM composite. Therefore, the MB-PACM resin is applicable for the carbon fiber reinforced polymers composites. Nal et al. [164] synthesized the vanillin/eugenol epoxy resin by curing the bio-based vanillin/eugenol curing agent (VECA) with a commercial epoxy matrix. The VECA wt% effect was evaluated on the thermal stability, impact resistance, and flexibility of the VECA epoxy resin. In comparing 100 wt% loading VECA, all coated materials did not show any cracks when the tape peeled off from the substrate. The cross-hatch test results suggested that the coating matrix applies to composites with good adhesion properties. Flexibility and impact resistance also suggested that all coated material resin could bear the mechanical force except for 100 wt% loading VECA. Due to the rigid structure of VECA and higher cross-link density, the pull-off adhesion property of composites decreased with the increasing wt% of VECA content compared to the commercial cured epoxy resin. Chu and coworkers [165] have recently reported that the flame retardant VN-based toughening curing agent, PVSi, for the epoxy matrix (EP). They studied the effect of PVSi on the impact toughness and FR. Relative to the neat epoxy, EP/PVSi-5 wt%’s impact strength reached up to 35.98 kJ/m^2^ from 12.42 kJ/m^2^ that increased may be due to the multifunctional groups of PVSi. Moreover, fracture surface roughness and surface irregular cracks increased with the incorporation of PVSi content, while the neat epoxy demonstrated the flat surface with straight lines cracks. The irregular cracks lead to higher surface energy and plastic deformation energy, resulting in higher toughness [166]. On the other hand, impact toughness decreased with the overused of PVSi in the composite due to the rigid structure [167]. Besides experimental methods, theoretical approaches are also essential to investigate the mechanical properties of natural fiber/polymer composites, and among them, fractal theory is an essential tool. Xiao et al. have presented a fractal analytical model for fluid transport through the roughened surface of the porous fibrous materials without using any empirical constant that was usually required in earlier models. Further, they proposed that this model may be used to investigate other transport properties, including gas diffusivity and thermal conductivity of porous fibrous materials, and may also be used for other porous materials other than fibrous materials [168,169]. The LOI value of the composite increased from 28% to 34% with the incorporation of wt% fraction of PVSi (Figure 13a), although EP/PVSi-5 wt% can pass the UL-94V0 test with the low phosphorus content. In the LOI test, the char residue increased with the increasing PVSi fraction (Figure 13b), and also UL-94V0 vertical burning test showed the same trend (Figure 13c). As shown in digital photos, the neat EP strip was completely destroyed (Figure 13d) compared to the EP/PVSi-5 wt% (Figure 13e). Moreover, EP/PVSi-5 wt% showed auto-extinguishable property without melting whereas neat epoxy exhibited the melting with dropping. In addition, the values of peak heat release test and the total heat release decreased with the increasing wt% of PVSi that means the composite showed the fire safety performance than those of the neat epoxy matrix.

**Figure 14:**
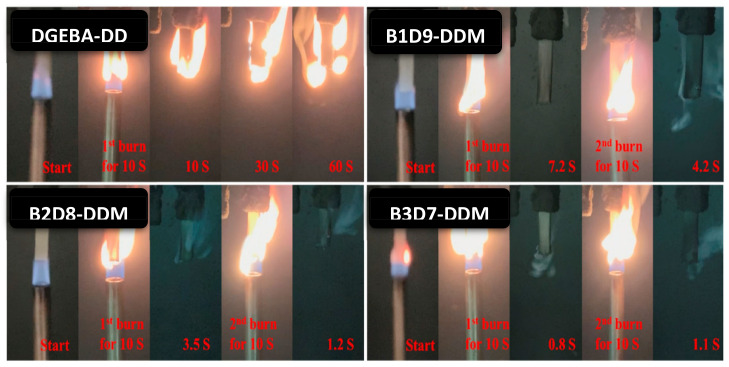
Digital photos of DGEBA-DDM, B1D9-DDM, B2D8-DDM, and B3D7-DDM for the UL-94 test. Reprinted with permission from J. Liu et al. [170] Copyright 2020 Elsevier.

Liu and coworkers [170] prepared the DGEBDB-DGEBA-DDM-based composites, in which the DGEBDB monomer was prepared from lignin-derived VN and guaiacol. The ratios 1:9, 2:8, and 3:7 of DGEBDB-DGEBA with curing agent DDM, B1D9-DDM, B2D8-DDM, and B3D7-DDM did not show intensive combustion while the DGEBA-DDM displays flammability (Figure 14). The self-extinguishing time of the composites decreased with increasing the content of DGEBDB with phosphorus. B3D7-DDM, with the phosphorus content of 1.81%, exhibited the shortest self-extinguishing time, 1.9 s. Meanwhile, the DGEBDB-based composite did not show any flaming drip from the burning samples. As increased the content of DGEBDB, LOI values increased from 24.2% to 33.4%, and the highest LOI value appeared for B3D7-DDM with the phosphorus content of 1.81%. This result demonstrated that the DGEBDB is a potential bio-based monomer to improve the epoxy matrix’s fire resistance. Recently, bio-based vanillyl alcohol (VA)/lignin-containing cellulose nanofibrils (LCNFs) have been developed [171]. They have used the VA epoxy resins to improve the compatibility with LCNFs. After loading the wt% of LCNFs, VA/LCNFs composites show a significant reinforcement effect. At 1 wt% of LCNFs, nanocomposite displayed the tensile strength 81% higher as compared to VA. Although after 1 wt% loading of LCNFs, the tensile strength decreased, which may be attributed to the compatibility and the aggregation. Moreover, toughness and strain energy density of nanocomposite increased with the loading of 1 wt% LCNFs. Despite the higher stiffness of LCNFs, the nanocomposite’s tensile modulus did not improve significantly, and it might be due to the less sensitivity to interfacial properties. Owing to the non-polar moiety of lignin, it can reduce the hydrophilicity of natural fiber cellulose. The resin showed a significant improvement in compatibility with lignin-based cellulose nanofibrils due to the interaction between epoxy matrix and fibrils [16]. The significant improvement in bio-based VA/LCNFs nanocomposite properties indicates that it can substitute petroleum-based epoxy resin in natural fiber composites.

## 7. Applications of Divanillin-Based Resin

DVN is known as a bio-based phenolic compound, and it is used in broad applications such as test enhancers, the food industry, organic light-emitting diode, and nanocomposite materials [121,122,172]. A few research studies have been published on the DVN-based materials, including one review paper [121,122,172,173]. Limited research has been published on the DVN-based epoxy thermoset resins and used as a DVN-based curing agent to prepare the thermoset materials [131,137]. Savonnet and coworkers [131] reported the bio-based DVN epoxy thermoset composites. The composites were prepared by mixing DVN-based epoxy monomers, namely, di-GEDVA, tri-GEDVA, and tetra-GEDVA, in curing agent, IPDA. The T_g_ of all composites (Di 60%-Tri 20%-Tetra 20%, Di 20%-Tri 60%-Tetra 20%, and Di 20%-Tri 20%-Tetra 60%) found to be comparable with DGEBA. In the FR test, GEDVA thermoset specimens stopped burning rapidly with the char layer while the DGEBA thermoset burned quickly with flame. DVN-based thermosets are promising materials for composites as flame retardant high performance and adhesive applications.

## 8. Conclusions

This review paper elucidated broad research literature and synthetic activities on lignin, VN, and DVN epoxy resins, focusing majorly on curing kinetics and curing agents, thermal stability, adhesion property, and mechanical properties. All recent aspects of lignin, VN, and DVN epoxy materials were reviewed, with perspectives for alternative toughened thermosets for natural fiber composites, FR, and as adhesives for high-performance application.

Although commercial epoxy resins are prevalent for various applications, bio-based epoxy resins from lignin, VN, and DVN could be alternates for commercial epoxy resins due to their high thermal stability tensile strength with desirable ecofriendly and environmental safety. Many papers have been published on the lignin, VN, and DVN related to coatings, FR, adhesives, building materials, and high-performance applications. However, limited study has been published on the lignin and VN-based epoxy resins for natural fiber composites, although bio-based DVN epoxy resins are flourishing for natural fiber composite applications. Additionally, DVN can also be devoted to developing bio-based curing agents to reduce environmental concerns. This review explored the utilization of lignin-derived bio-based resin to develop natural fiber/polymer composites with high-performance applications.

## 9. Future Prospective

With more attempts from the worldwide research community, advanced flame retardant and high-performance lignin, VN, and DVN-based epoxy resins might be a technical and commercial success for natural fiber composite applications. This review will increase the research community’s attention to bio-based resins production to reduce the use of petroleum-based resins. The literature depicts limited research progress on resin’s adhesion strength with natural fibers, including natural fiber composites development with lignin-derived resins. However, this literature shows challenges that must be overcome to develop fully bio-based resins, including bio-based hardeners, and enhance resin’s binding adhesion strength with natural fibers to obtain environment-safe high-performance composites. Finally, we are optimistic that combining various tailoring properties and the design methodology will achieve technical and commercial success. Nevertheless, there will be a massive need to improve the resins’ adhesive property and toughness for natural fiber composites.

## Figures and Tables

**Table 1:** A summary of thermal and mechanical properties of the lignin-based network.

Lignin Based Network	Solvent	T_g_ (°C)	T_s_ (°C)	IDT (°C)	Char Residue (%)	Tensile Strength (MPa)	Young’s Modulus (GPa)	Toughness (kJ/m^3^)	Ref.
DKL-DDM	-	-	179	290	38	-	-	-	[56]
DOL-DDM	-	-	166	257	29	-	-	-	[56]
DKL-DETA	-	-	165	252	33	-	-	-	[56]
DOL-DETA	-	-	148	228	23	-	-	-	[56]
DLEP	-	-	-	258	-	2.66	-	-	[65]
OLEP	-	-	-	244	-	-	-	-	[65]
LHEP/BADGE1:1	-	80	161	-	-	-	-	-	[72]
LHOEP/BADGE1:9	-	111	169	-	-	-	-	-	[72]
X-40LB	Dioxane	-	151	-	30	34.3 ± 6.2	-	-	[75]
PVA-lignin blend	DI water	-	-	230	-	41.1 ± 4.2	2.48 ± 0.10	0.6 ± 3.2	[35]
PEO-lignin blend	Methanol aqueous solution	-	-	350	22.5	6.2	0.28	-	[31]
EPLM	DI water	-	-	200	-	48.5 ± 6.2	2.4 ± 0.2	0.787	[34]
GL-PGEDGE	-	-	-	-	-	5.0	0.08	-	[76]

**Table 2:** A summary of thermal and mechanical properties of VN epoxy-based resins.

VN Epoxy-Based Network	T_g_ (°C)	T_d5_ (°C)	Char Residue (%)	Tensile Strength (MPa)	Young’s Modulus (MPa)	FR	LOI (%)	Ref.
DGEMHQ	132	-	20	-	-	-	-	[90]
DGEVAC	152	-	14	-	-	-	-	[90]
DGEVA	97	-	19	-	-	-	-	[90]
VNER/EF205	38.3	-	-	14.86 ± 1.9	2011.63 ± 49.3	-	-	[33]
VNER/EF205/2 wt% IA	47.9	-	-	30.58 ± 1.7	959 ± 27.6	-	-	[33]
DGEBA/EF205	52.1	-	-	29.63 ± 1.3	1770.88 ± 46.4	-	-	[33]
DGEDVCP-PN	-	394	59	-	-	-	-	[98]
DGEDVCP-QC	-	386	61	-	-	-	-	[98]
DGEDVCP-GCN	-	387	48	-	-	-	-	[98]
EP1-DDM	183	340	53	80.3 ± 5	2114 ± 132	√	31.4	[8]
EP2-DDM	214	353	58	60.6 ± 3	2709 ± 110	√	32.8	[8]
DGEBA-DDM	166	382	14	76.4 ± 6	1893 ± 140	˟	24.6	[8]
TE0-APP15	153	353	28	-	-	√	35.4	[108]
TE10-APP0	176	342	26	-	-	˟	32	[108]
MP1.5-APP3.5	176	355	25	-	-	˟	25	[108]
MP3-APP7	164	348	23	-	-	√	29.4	[108]
PLA/5VP	-	325	0.3	54 ± 1	3500 ± 100	√	25.8	[88]
PLA/5VP	-	324	0.7	52 ± 1	3500 ± 200	√	26.3	[88]

**Table 3:** Adhesion strength performance of lignin-based resins.

Resin	Substrate	Shear Strength (MPa)	Ref.
PVA-Lignin	CNFs	4.4	[35]
PEO	CNFs	0.442	[31]
30% PEO-L	CNFs	0.865	[31]
EPL40	CNFs	6.8	[34]
50% DOL-DDM	Stainless steel	7.7 ± 0.3	[155]
KL-GDE	Birch plywood	2.1	[156]
